# Accessibility of Motion Capture as a Tool for Sports Performance Enhancement for Beginner and Intermediate Cricket Players

**DOI:** 10.3390/s24113386

**Published:** 2024-05-24

**Authors:** Kaveendra Maduwantha, Ishan Jayaweerage, Chamara Kumarasinghe, Nimesh Lakpriya, Thilina Madushan, Dasun Tharanga, Mahela Wijethunga, Ashan Induranga, Niroshan Gunawardana, Pathum Weerakkody, Kaveenga Koswattage

**Affiliations:** 1 Faculty of Technology, Sabaragamuwa University of Sri Lanka, Belihuloya 70140, Sri Lanka; kaveendra@tech.sab.ac.lk (K.M.); kmchamarakumarasinghe@gmail.com (C.K.); ashaninduranga@tech.sab.ac.lk (A.I.);; 2 Faculty of Computing, Sabaragamuwa University of Sri Lanka, Belihuloya 70140, Sri Lanka; jirandika@std.appsc.sab.ac.lk; 3 Faculty of Applied Sciences, Sabaragamuwa University of Sri Lanka, Belihuloya 70140, Sri Lanka

**Keywords:** motion capturing, cricket batting, computer vision, coaching, machine learning

## Abstract

Motion Capture (MoCap) has become an integral tool in fields such as sports, medicine, and the entertainment industry. The cost of deploying high-end equipment and the lack of expertise and knowledge limit the usage of MoCap from its full potential, especially at beginner and intermediate levels of sports coaching. The challenges faced while developing affordable MoCap systems for such levels have been discussed in order to initiate an easily accessible system with minimal resources.

## 1. Introduction

**Figure 1:**
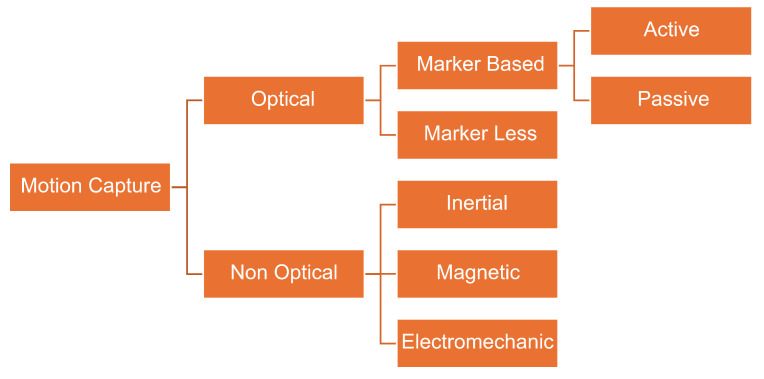
Classification of motion capture technology.

Motion capture, often abbreviated as **“MoCap”**, is a technology that uses sensors or markers placed on the body to track and digitally replicate human movements. Incorporating motion capture has transformed how movements are studied, analyzed, and reproduced across different fields. Motion Capture (MoCap) has been extensively used in various fields of research and industrial applications such as animation, filmmaking, video games, sports, and medical research [1,2]. In MoCap, the movement of humans and objects is recorded. High-end instrumentation and sophisticated computer algorithms, particularly those utilizing deep learning techniques, have significantly propelled the advancements in MoCap technology [3,4]. Motion capture is usually performed using two methods (as illustrated in Figure 1). These methods specifically include non-optical and optical approaches. In the case of optical systems, despite their higher processing cost, they provide excellent precision and total freedom of movement. Additionally, they offer the potential for interaction between diverse subjects [5]. Camera-based optical systems are categorized into two subcategories: marker-based systems and marker-less systems. A comprehensive review of these aspects and uses of MoCap in industrial applications has been discussed elsewhere [6]. Moreover, the use of MoCap in sports is steadily increasing with time, opening up many research opportunities at all levels of study. To drive this forward, new and efficient algorithms and libraries are required.

High-speed cameras and advanced position-sensing sensors are important for motion capture systems. However, making these systems more affordable and accurate could make them more useful in many different areas. Our group has recently initiated the development of such a system specifically focused on cricket. It is absolutely essential to conduct a comprehensive review of the existing technology landscape in order to gain a proper understanding of the accessibility and availability of current systems. This review serves as a crucial step in exploring how MoCap technology can revolutionize the analysis of cricket batters and bowlers, offering an in-depth exploration of its potential impact.

## 2. MoCap in Medicine

Gait analysis in medical treatments has made substantial use of motion capture technology. It is common practice to utilize inertial sensors in this area. Cloete in his study compared the “Moven” inertial motion capture system against the **“Vicon”** motion capturing (Vicon Motion Systems Ltd., Oxford, UK) system in gait analysis revealing that both systems were providing accurate and consistent results [7]. Pfister and Co. studied the comparative uses of **“Kinect”** (Microsoft Corporation, Redmond, WA, USA) and Vicon 3D (Vicon Motion Systems Ltd., Oxford, UK) in gait analysis. Three-dimensional systems like Vicon are considered to be much more accurate and reliable in clinical settings. However low-cost alternatives to measuring gait timing and alignment are proposed to be vital. Usually, these include two-dimensional (2D) video cameras with analytic software, electrogoniometers, pressure-sensitive mats, or accelerometers. The Kinect sensor released by Microsoft used an infrared (I.R.) light protector, an I.R. camera, and an RGB video camera. They concluded that the Kinect has basic MoCap capabilities and using them in clinical settings may need thorough and careful modifications in both software and hardware [8].

Recent studies show that sound feedback called “motion sonification” can help people with stroke rehabilitation. This method uses sounds to tell them how they are moving, like when they sip water during their rehab exercises [9,10]. Bruckner et al. implanted an inertial sensor system consisting of a wireless sensor network comprising a maximum of 10 inertial sensors. These sensors are securely attached to the patient’s body to accurately capture complex upper body movements, as well as force finger sensors that can recognize gripping motion. Orientation estimation allows the use of forward kinematics and a model of rigid links connected by joints to compute the positions, angles, and speeds of the upper limbs, which is based on information from an inertial measurement unit (IMU) installed on the upper arm and forearm. The wireless IMU stands out for its low-power RF module and 8-bit Main Control Unit (MCU), allowing for full access through platform-independent interfaces. Additionally, it offers real-time onboard orientation estimation, making it a valuable tool [11].

Miniaturization of surgical tools and the creation of better equipment for manipulating specialized tissues, in particular, are the main driving forces behind the reduction in surgical trauma and the development of minimally invasive surgery (MIS) [12]. Through the use of a trocar port (a trocar port refers to a cylindrical cannula that houses a trocar, a medical device with a pointed tip, used for the purpose of introducing ports into the abdominal cavity), optical fibers or light-emitting diodes, and either a body-mounted complementary metal oxide semiconductor sensor or a digital video feed, MIS allows the surgeon to reach the operative site and illuminate it. A laparoscope with lenses and an external camera are attached [13]. On a similar topic, Laribi and coworkers proposed a slave robotic architecture for compact MIS. They highlighted the emulating the complex movements required while creating a surgical connection between two structures [14].

Robot-assisted MIS can reduce hand tremors by using scaling factors and extra joints in the robotic tools that match the surgeon’s hand movements. This can improve the surgical skills and outcomes of the patients and enable more complicated surgeries. However, most of the existing robotic surgical systems are too big, complex, and expensive to use widely. They also take significant time to set up, maintain, and sterilize. A simple, small, and portable robotic surgery system that has similar performance, easy usage, high adaptability, and quick setup time can increase the use of mechanical assistance in regular and other types of surgeries. The da Vinci Surgical System [15] and the ZEUS System [16] are some of the current well-known automated systems that assist surgeons in MIS operations.

In physiotherapy and diagnostics, many people are interested in using motion capture devices that can sense human movement and gestures in real time without touching the body. In a study conducted by Yuminaka et al., they assessed the feasibility of MoCap applications in medical and healthcare using a Kinect v2. The real-time motion capture and depth measuring capabilities of Kinect sensor was assessed to be used in non-contact visual data observation while calculating distances between objects with spatial resolution on the order of millimeters. They suggested this may help therapists and patients with diagnosis and rehabilitation [17].

Apart from rehabilitation, biomechanical analysis MoCap is currently used in surgical planning [18], virtual surgical training [19], patient education [20], and telemedicine [21].

## 3. MoCap in Sports

Motion capture analysis for sports uses specialized technology to capture and analyze athletes’ movements. MoCap analysis is a powerful tool that can be used to improve the performance of athletes and reduce the risk of injuries [22]. For example, coaches can use motion capture data to see how an athlete’s running gait compares to elite athletes [23]. In the kinematics analysis, MoCap data can be used to measure the athlete’s joint angles, velocities, and accelerations. This information can be used to identify areas where the athlete’s technique can be improved [24]. The kinetics analysis can also be used to measure the forces and torques that the athlete is producing. This information can be used to assess the athlete’s strength and power, identify areas where they can improve their output, and measure the athlete’s energy expenditure [25]. This information can be used to assess the athlete’s fitness level and identify areas where they can improve their efficiency and performance.

Regarding sports injury prevention, the MoCap analysis can be used to identify athletes at risk of injury by identifying movement patterns associated with an increased risk of injury. If athletes have been identified as being at risk of injury, motion capture analysis can be used to develop personalized training and injury prevention programs. This technology can also track the athlete’s progress during injury prevention programs [26]. In addition to the above, MoCap can be used to (i) develop new and innovative training methods that are designed to reduce the risk of injury, (ii) improve the design of sports equipment to make it safer for athletes, and (iii) develop more effective rehabilitation programs for athletes who have been injured [27].

Motion capture technology has been widely used in baseball for various purposes. MoCap helps understand pitching mechanics, including the release point, arm angle, and body posture. This information is valuable for pitchers and coaches, allowing them to adjust for better performance and reduced stress on the player’s arm [28]. Improving hitting abilities requires careful analysis of baseball players’ swing mechanics. MoCap offers information on a swing’s body motions, bat speed, and angle. This information assists in honing methods and streamlining the striking procedure [29]. It may also be used to spot behaviors or trends that could result in harm. Players may concentrate on improving their technique by identifying dangerous actions, and coaches can create specialized conditioning regimens to stop injuries.

In swimming, there are two main types of MoCap systems: optical and inertial. Optical systems use cameras to track reflective markers placed on the swimmer’s body. Inertial systems use sensors attached to the swimmer’s body to measure movement. Most swimmers use more wearable sensors than other MoCap technologies because wearable sensors are small in size, cheap in price, and free from site restrictions. They used MoCap systems to analyze the biomechanics of swimming strokes (freestyle, breaststroke, backstroke, and butterfly), identify areas where swimmers can improve their technique, develop new training methods that are more effective and less likely to cause injury, as well as create realistic swimming animations for video games and movies [30].

Human movements and action analysis are significant to karate. They can improve karate techniques, such as punches, kicks, and blocks, using the MoCap analysis system [31]. Karate requires physical, technical, and tactical skills to succeed in the sport. These techniques depend on how the foot, knee, or elbow is moved. Currently, athletes and coaches are working to improve the sport of karate using MoCap systems [32].

## 4. Other Fields

Various fields beyond the ones mentioned utilize motion capture technology, including the construction industry [6], entertainment industry [33], virtual reality and augmented reality [34], automotive industry [35], aerospace and defense [36], forensics and law enforcement [37], education [38], etc.

Han et al. carried out an empirical assessment of an RBG-D sensor motion capture and action recognition for monitoring construction workers [39]. Using stereo cameras, Richmond and his team were able to capture the motion of a construction worker, with kinematic modeling to evaluate productivity and safety [40]. Seo et al. studied motion capture approaches for body kinematics measurements in construction to reduce significant unsafe environmental and health risks [41].

In 1915, Max Fleischer developed a technique to give their characters a realistic fluid motion in animations, which can be debated as the first attempt at motion capturing [42]. Zeeshan et al. executed a study about real-time motion capture for the entertainment industry. They developed the “eMoveChat” application, which can imitate video, voice, and trigger events using the **“Animazoo”** motion capture system (Animazoo UK Ltd., East Sussex, UK) [43]. Robin et al. introduced collaborative Virtual Reality (V.R.) with MoCap to enhance the entertainment industry in poor acting conditions. That collaboration made it easier for film and video game directors to digitize the characters [44]. Knyaz studied a photogrammetric motion capture system “MOSCA” to collect accurate 3D motion object information. The MOSCA method provided highly accurate and reliable information [45]. Chan and the team used MoCap with virtual reality to implement a system for practicing the Chinese martial art Tai Chi [46].

Kim et al. used the MoCap to study the autonomous formation flight of multiple flapping-wing flying vehicles for the first time as the turning point of aviation [47]. Wu et al. provided a technique for evaluating maintainability of a civil airplane using an optical motion capture system. After that, a test case was created to show that the approach was workable. The test case primarily addressed the civil aircraft’s accessibility [48].

Phunsa et al. performed a study introducing a Thai boxing defense system using MoCap using nine optical motion capture cameras with 42 markers for two actors [49]. Parks et al. developed a portable mobile motion capture system, “${\mathrm{MO}}^{2}$CA”, for military use in rural environments and clinical situations [50].

Joint angles of the human body are important identification features in forensic science. So Yang et al. investigated the use of marker-less motion capture systems for person tracking in forensic biomechanics applications [37]. To investigate the dubious forensic cases Aquila et al. explored the reconstruction of the dynamics of a murder using 3D motion capture and 3D modeled buildings [51].

In the education field, the collaboration of virtual reality and motion capture can fill the gap between virtual classrooms and real classrooms, according to research by Alonso et al. considering secondary school teacher training [52]. Yokokohji et al. studied a method to teach a robot using motion capture from the demonstrator’s viewpoint [53].

## 5. Cricket-Related Literature

As our focus is on cricket, it is important to look at the efforts that have been made by others to enhance the performance of cricket skills by MoCap. So we will now discuss it in two subsections, namely bowling and batting.

### 5.1. Cricket Bowling

MoCap has gained popularity in cricket bowling analysis in the last several years. Real-time tracking of the ball’s and a bowler’s joint movements is possible with MoCap systems. These data can then be used to analyze the bowler’s technique, identify areas for improvement, and reduce the risk of injury. Cricket bowlers are limited to 15 degrees of elbow extension during the bowling action [54]. This complex movement requires 3D motion analysis to be assessed accurately. Traditionally, this has been achieved using marker-based motion capture systems in a laboratory setting. However, this raises concerns about ecological validity, as cricket bowlers typically train and compete outdoors. Researchers are now developing new methods for 3D motion analysis that can be used in outdoor settings. This will allow for more accurate and realistic assessments of cricket bowlers’ technique, which could lead to improved performance and reduced risk of injury [55]. Researchers have developed a wearable arm sensor that can be used to assess a bowler’s bowling action in real time. This technology could help umpires make more accurate decisions about whether or not a bowler is using an illegal throw-like bowling action. The sensor uses inertial sensors on the upper and lower arms to track the bowler’s movements. Suspicious deliveries reveal valid distinctive inertial signatures. The technology is still under development, but it has the potential to revolutionize the way that bowling actions are assessed in cricket. It could also be used as a training tool for developing bowlers [56].

Kumar and his group created a cricket ball with magnetometers that calculated the spin rates at high speeds and identified the spin types (off-spin and leg-spin). The spin type is validated by IMUs on the bowler’s wrist and elbow. The researchers proposed that the magnetometer and the IMU sensors can help customize training and track performance. This technology has the potential to revolutionize the way that spin bowling is analyzed and trained. It could help bowlers improve their technique and consistency, and it could also help coaches identify and address deficiencies in bowlers’ techniques [57]. Coaches can use the findings of the study to identify key performance indicators (KPIs) for spin bowlers. By tracking these KPIs, coaches can assess the progress of their bowlers and develop training programs that help them to improve their performance. Players can also use the findings of the study to identify areas where they can improve their bowling techniques using MoCap systems [58].

Wells et al. also developed a predictive model to predict ball deviation based on the four most important kinematic variables: average velocity, elbow joint angle, angle of release, and ankle joint angle. The regression equation was reliable and explained 97.4 percent of the total variability in ball deviation [55].

Harnett and Co. tested an array-based IMU to measure cricket fast bowling movements as a first step to see if it could be used for tele-sport and exercise medicine. They found that the IMU-based system can measure specific cricket fast bowling movements accurately, such as the angle between the shoulder girdle and the pelvis, the bending of the trunk, and the bending of the knee. Thus, they speculated that the IMU-based system can help identify injury risk in tele-sport and exercise medicine [59].

In another study carried out by Ferdinands et al., they examined how rear leg movements and wrist speed influenced fast bowling. It revealed that bowling wrist speed was associated with several factors, such as the average speed of thigh extension, the speed of thigh adduction at the moment of back foot contact, and the maximum change in speed of knee extension. The study also showed that rear leg drive was not a deliberate action, but rather a passive outcome of the hip and knee movements in different planes, which had minimal and regulated effects on torque motion. The study used a Cortex 2.0 motion analysis system (200 Hz) to measure these variables [60].

### 5.2. Cricket Batting

Using motion capture and computer vision techniques, it is possible to extract precise information regarding player movements and the paths of the ball. This information has the potential to contribute to the advancement of knowledge in the field of cricket performance. For instance, computer vision techniques can be employed to identify the crucial technical factors that contribute to a batsman’s success [61].

Moodley et al. tested the potential of deep learning architectures (AlexNet, Inception V3, Inception Resnet V2, and Xception) to identify cricket batting backlift techniques in video footage. It showed that deep learning architectures could reliably detect cricket batting backlift techniques in video footage, with the “Xception” architecture achieving the highest accuracy of 98.25 among the four architectures evaluated [62].

In 2016, Peploe’s study explored how technique and bat speed affected post-impact ball speed and carry distance in a cricket range hitting task. The study used an 18-camera Vicon motion analysis system and three high-speed video cameras to measure these variables. The study discovered that a large carry distance required a launch angle close to 42 degrees and a high launch speed. It also revealed that a high ball launch speed depended on an impact location near the bat’s sweet spot in both the horizontal and vertical directions, as well as a high bat speed [63]. In a previous study conducted in 2012, researchers found that bat speed plays a crucial role in achieving the necessary distance for hitting a six in cricket. They also discovered that hitting the sweet spot of the bat maximizes the power transferred to the ball [64].

In a 2019 study, researchers employed utilizing motion capture data, and machine learning algorithms demonstrated a remarkable capability in accurately predicting the trajectory of the ball. The neural network algorithm was the most accurate algorithm, with a mean absolute error of 1.5 degrees. This suggests that machine learning algorithms could be used to develop new tools and techniques that might help cricket batsmen and bowlers improve their performance [65]. In their study using the Vicon motion analysis system, researchers were able to assess the influence of both the impact of anthropometric characteristics (height, weight, and body composition) and technical variables (bat speed, launch angle, and impact location) on the batting performance of elite cricketers. Bat speed was the most important variable in predicting batting performance, followed by launch angle and impact location. Anthropometric characteristics were not found to be significant predictors of batting performance.

Efforts made by Callaghan et al. with motion capture analysis has revealed the rolling cricket start is the most effective way to accelerate during a quick single, and that cricketers should be aware of the kinematic alterations that occur when carrying the bat during a quick single [66].

Also, the study revealed higher back-lift was detrimental to bat alignment and that the batter with the highest back-lift showed the least bat control. They also found that on-side defensive strokes could be discriminated from the off-side defensive strokes by observing a significantly lower magnitude acceleration at ball contact [67].

In 2023, Siddiqui et al. used a novel approach employing motion capture in “MediaPipe” to extract features from cricket stroke videos and machine learning models to classify the strokes. They achieved an accuracy of 99.77 percent using a random forest model [68]. Stuelcken et al. employed an innovative method using two synchronized high-speed video camera motion capture systems to show that the batsmen used a unitary upper limb movement, that is, their arms and shoulders moved together as a single unit. This helped them to generate more power and control. The batter stepped forward with their front foot very late, just before hitting the ball. Their front ankle was pointed towards the inside of the field, which helped them to hit the ball with more power [69].

In 2016, Dhawan et al. conducted a pioneering study, capturing the movements of elite cricket bowlers using advanced motion capture technology. These data were then utilized to create virtual animations, meticulously replicating the trajectories of bowled balls. The researchers further developed an interactive virtual environment using Unity software, enabling users to engage with the simulation via a head-mounted display and a motion-tracked cricket bat [70]. Curtis et al. used a system that captured the motion of cricket batters while playing strokes and compared it to reference values set by coaches. Fuzzy sets were used to classify the cover drive and forward defensive strokes based on ranges of motion for the head, feet, and bat. The system provided feedback on how well the strokes were executed compared to expert batters [71].

Kelly et al. developed a motion capture system to analyze cricket batting strokes. The system records the movements of players performing strokes and compares them to benchmarks set by coaches. Using fuzzy logic, the system classifies strokes like cover drives and forward defenses based on movements of the head, feet, and bat. The system then provides feedback by evaluating the player’s technique against the standards of expert batters [72]. In their study, they explored both vision-based and depth-based motion capture techniques intending to integrate these technologies into a comprehensive system. They aimed to develop a framework for motion and gesture recognition that works well under different conditions. Minimizing latency and improving accuracy were also important design objectives. The proposed system’s innovative aspect was its ability to generate a real-time motion-captured avatar, which could potentially enhance user immersion and sense of presence.

Callaghan et al. conducted a detailed study that utilized the Vicon motion capture system to compare the effects of two different starting techniques, namely rolling start and static start, on cricket sprint performance metrics like sprint velocity and acceleration kinematics during a quick single maneuver by experienced cricket players. The research provides valuable insights to optimize cricket sprint strategies [73].

## 6. Cost-Effective MoCap Systems

There are plenty of researchers who are trying to introduce a low-cost MoCap method to analyze motion owing to the significance they possess, as we discussed above. Thewlis et al. used the **“OptiTrack”** system (Natural Point, Oregon, USA) as the low-cost system and Vicon MX-f20 system (icon Motion Systems Ltd., Oxford, UK) as a high-cost system and obtained a comparison of lower limb gait kinematics from each system [74]. Regazzoni et al. measured the performances and limitations between Sony PS Eye cameras and the Microsoft X-Bod Kinect RGB-D sensors to find a cost-effective method for MoCap [75]. Chatzitofis and co. introduced “DeMoCap” as the first data-driven method with marker-based MoCap using consumer-grade infrared-depth cameras. They used four Intel RealSense D415 sensors as marker-based systems and 24 Vicon MXT40S cameras as marker-less methods to compare them. In this study, they introduced their marker-based system as a low-cost method [76]. Conforming to the low-cost MoCap approach, Sgro et al. carried out a study to assess the vertical jump development levels in childhood using a Microsoft Kinect [77]. Robert et al. sorted out three comparisons to validate a low-cost Inertial Motion Capture (IMC) system for whole-body motion analysis. The first comparison was of Optical Motion Capture (OMC) vs IMC with both anatomical models to depict technological errors; second was the anatomical model vs Neuron model with both using IMC to render the kinematic model differences; and the comparison was finally settled by OMC using the anatomical model vs IMC using the Neuron model to illustrate the total difference [78]. Usually, 3D motion capturing needs multiple cameras to detect 3D behavior. This is indeed a high-cost method, albeit Lee et al. used a single camera and passive optical markers with low cost to capture the 3D motions. They used 3D localization of markers using monocular vision [79]. Choe et al. manifested a MoCap mechanism using Magneto-Inertial Measurement Unit (MIMU) sensors. They used MIMU’s 3-axis accelerometer, 3-axis magnetometer, and a compass to produce a novel calibration model [80]. Patrizi et al. executed a comparison of kinematics multipliers computed between Microsoft Kinect just as marker-less and the BTS SMART optical marker system [81]. To introduce a low-power and low-cost hand motion capture device Sama et al. built a glove as a Tri-dimensional Interaction Device (3dID), which was the gateway to identify gestures [82]. Raghavendra and others created a human character in Blender and controlled it using wireless sensor nodes located on each joint in the human body at a low cost. This sensor node included Wi-Fi SoC (ESP8622-12E), which communicates with the accelerometer (ADXL345), magnetometer (HMC5883L) using I2C protocol, and gyroscope (L3G4200D) [83]. Despite many attempts to create low-cost MoCap systems, with the current study we want to highlight that the low-cost term has been used comparatively. Several thousand dollars are sometimes considered to be low-cost against several hundred thousand dollars. We would like to emphasize the requirement of defining the low-cost term and what minimum features can be made accessible to bottom-layer users like college students.

## 7. Summary of the Literature

It can be seen that marker-based optical MoCap is the most common method used in many instances. This is largely due to the accuracy that can be achieved. However, hefty cost is unavoidable. It is expensive because (i) accuracy depends on the number of cameras used, (ii) the system requires complex setup, and (iii) placing markers requires careful attention. Inertial sensor-based motion capture is the other popular option. They usually consist of accelerometers and gyroscopes. The main advantage of IMUs is that they do not require expensive camera systems. However, using them in harsh environments, like sports, may pose some difficulties. But if one can eliminate them, this can be a good alternative to marker-based optical systems. From another point of view, marker-less systems can be a good alternative to expensive commercial systems. Currently Microsoft Kinect, Apple ARkit, and Google ARCore are some upcoming technologies for marker-less systems. These are ideal where the measuring times are long and the outcome requires continuous acquisition of many players without having to worry about implanting foreign objects such as markers and wired sensors on the player. Industrial giants like Vicon, Qualisys, and Optitrack also have marker-less solutions. The quality of marker-less systems depends on the algorithms that have been used. There are also libraries like OpenPose and Mediapipe that are popular in the cases of custom systems. OpenPose gives the user the capability of (i) real-time performance, (ii) multi-person capability, and (iii) versatile keypoint detection. Similarly, Mediapipe also includes a variety of pre-trained machine learning models, for tasks like face detection, hand tracking, and pose estimation and also can perform efficiently in real time. (For a quick overlook refer to Table 1) Other than those listed, PoseNet [84], DeepMotion [85], AlphaPose [86], ZED SDK by Sterolabs [87], and SimplePose [88] also have been used in MoCap quite effectively.

In sports like tennis, baseball, and golf, marker-based systems are widely used in controlled environments like training centers for precise biomechanical analysis. Athletes in fast-moving sports like basketball and football benefit from marker-free systems. Outdoor and extreme sports like skiing and cycling use inertial and magnetic sensors to measure movement dynamics without cameras. Due to their restrictions on natural movement, mechanical methods are less common but used to measure force and mechanical efficiency in cycling. When it comes to cricket, marker-based systems are used in training scenarios to analyze batting and bowling techniques. High precision is required for detailed biomechanical feedback. Marker-less systems can be useful during live games or in practice sessions to track player movements without intrusive markers, particularly for fielding analysis and running between the wickets. Inertial sensors are employed mainly in training to gather data on player movements and biomechanics without the need for a full optical setup. Mechanical methods could be used experimentally to measure the force of impact in batting and the mechanics of bowling (refer Table 2).

## 8. Conclusions

Based on our thorough review of the existing literature, we aim to draw valuable insights that will shape the upcoming work outlined in the grant mentioned in the funding section. We expect to analyze and enhance batting skills for beginner and intermediate-level players. Hence, we will have to think about the cost of our system. Computer vision and machine learning techniques are incredibly useful for creating affordable motion-capturing systems. Utilizing software-based processing methods can significantly decrease the expenses associated with using costly hardware but inherits challenging tasks like calibration of camera networks and data validation of using commercial setups [96,97]. Nevertheless, ensuring that the footage remains clear and stable may prove to be quite challenging. Also, we have to make sure that we are using algorithms that can resolve both indoor and outdoor setting efficiently. This puts the balance towards using high frame-rate cameras as the input devices. Another aspect of decision making in the following steps of the project is to define the scope of interest, such as either kinematics of a specific angle or study of angular velocities. In the literature, most of the works have been performed by constraining the domain of analysis to either several strokes or several poses. We will be trying to compare different types of angle dynamics of professional players and novice/intermediate players generated by a homemade system and two commercial setups. By this we can elucidate the potential of low-cost system to assist coaches with more overall analysis.

## Figures and Tables

**Table 1:** Quick overlook of existing MoCap systems.

Method	Accuracy	Setup Complexity	Cost	Environmental Needs	Prior Usage
**Optical (Marker-based)**	Very accurate; captures detailed movements.	Complex setup; needs many cameras and careful placement of markers.	Expensive; requires lots of cameras and computer power.	Needs controlled lighting and clear view of markers.	[5,18,26,44,45,46,51,60,66,70,73,76,89,90,91]
**Optical (Marker-less)**	Very accurate; quality depends on software.	Medium-to-complex setup; depends on software.	Medium to expensive; needs advanced software.	Needs clear view of the person, sensitive to surroundings.	[1,20,22,35,76,92]
**Inertial**	Quite accurate; might lose accuracy over time.	Easy-to-medium setup; no external cameras needed.	Medium cost; sensors and processors are needed.	Very flexible; works anywhere, but needs initial setup.	[2,7,11,28,31,59,76,80,93]
**Magnetic**	Fairly accurate; can be disrupted by metals.	Medium setup; involves placing magnetic sensors.	Medium to expensive; specialized equipment needed.	Must avoid metal in the area.	[71,80]
**Mechanical**	Quite accurate; measures movement at joints directly.	Medium setup; involves wearing a suit with sensors.	Medium cost; suits and sensors can be pricey.	Suit needs to fit well; can limit movement.	[94,95]

**Table 2:** Usage of differentmotion capturing method in sports.

Sport	Marker-Based Optical	Marker-Less Optical	Inertial & Magnetic	Mechanical
**Golf**	⊗			
**Baseball**	⊗			
**Gymnastics**	⊗	⊗		
**Diving**	⊗	⊗		
**Basketball**		⊗	⊗	
**Football (Soccer/American)**		⊗	⊗	
**Athletics**		⊗	⊗	
**Cycling**			⊗	⊗
**Skiing**			⊗	⊗
**Rowing **			⊗	
**Canoeing**			⊗	
**Tennis**	⊗			
**Mixed Martial Arts**	⊗	⊗	⊗	
**Boxing**	⊗	⊗	⊗	
**Cricket**	⊗	⊗	⊗	

⊗ represents the abundance of usage of each method in particular sport.
